# The Human Exposome: Integrating the Environment, Human Health, and Society for the Next 60 years

**DOI:** 10.1021/acs.est.6c03080

**Published:** 2026-05-05

**Authors:** Pablo Gago-Ferrero, Alexandria B. Boehm, Heileen Hsu-Kim, Xiang-Dong Li, Jacqueline MacDonald Gibson, Martine Vrijheid, Bin Wang, Julie Zimmerman

**Affiliations:** † 203229 Institute of Environmental Assessment and Water Research (IDAEA-CSIC), Barcelona 08034, Spain; ‡ Department of Civil and Environmental Engineering, 6429 Stanford University, Stanford, California 94305, United States; § Department of Oceans, Stanford University, Stanford, California 94305, United States; ∥ Department of Civil and Environmental Engineering, 3065 Duke University, Durham, North Carolina 27708, United States; ⊥ Department of Civil and Environmental Engineering, 26680 The Hong Kong Polytechnic University, Hung Hom, Kowloon, Hong Kong, China; # Department of Civil, Construction, and Environmental Engineering, 5755 North Carolina State University, Raleigh, North Carolina 27695, United States; ¶ Barcelona Institute for Global Health (ISGlobal), Doctor Aiguader 88, Barcelona 08003, Spain; ∇ Institute of Reproductive and Child Health, School of Public Health Peking University, Beijing 100191, P.R. China; ○ Key Laboratory of Reproductive Health, National Health and Family Planning Commission of PR China, Beijing 100191, China; ⧫ Department of Chemical and Environmental Engineering, Yale University, New Haven, Connecticut 06511, United States; †† Yale School of Forestry and Environmental Studies, Yale University, New Haven, Connecticut 06511, United States

**Keywords:** exposome, biomonitoring, microbiome, environmental epidemiology, environmental justice, precision health, prevention

## Abstract

The exposome framework promises comprehensive characterization of chemical, physical, and biological exposures shaping human health, yet the measurement capacity now vastly outpaces interpretation and action. Here, we synthesize emerging frontiers that define the translation of exposome science into prevention: moving from “chemical dark matter” in high-resolution mass spectrometry toward functional exposomics; integrating the microbial exposome as both the target and modulator of exposures; deploying AI-enabled causal inference to bridge molecular precision with population-scale patterns; and embedding exposome evidence into proactive interventions, green chemistry, environmental redesign, and environmental justice frameworks. Progress over the next six decades will depend not only on measurement comprehensiveness but also on our capacity to shift from documenting environmental harm to designing healthier environments.

## Graphical abstract

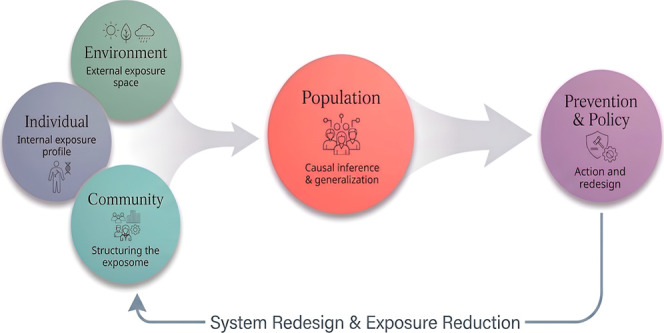

## Introduction: From the Environment to Human Health

*The environment is not only outside us; it is within us.* Over the past few decades, environmental science has progressively shifted from describing contamination in air, water, and soil to measuring and understanding how environmental factors become biologically embedded in the human body. This transition reflects a broader recognition that exposures are not external events but dynamic processes that interact with human biology throughout the life course. The concept of the human exposome has emerged as a natural evolution from this perspective. By encompassing the totality of chemical, physical, biological, and nutritional exposures experienced from conception onward, the exposome provides an integrative framework to study how environments shape human health over time.^1^ Importantly, it moves beyond single stressors, emphasizing cumulative, interacting, and context-dependent exposures that better reflect real-world conditions. This shift from reductionist to integrative thinking represents not only a technical advancement but also a fundamental reconceptualization of how we understand environment–health relationships.

Today, exposome research brings together disciplines that have traditionally evolved in parallel. Advances in environmental chemistry and exposure assessment technologies now allow increasingly comprehensive characterization of both internal and external exposures,^2^ while new insights from microbiological sciences have illuminated the dual roles of microbiomes as modulators of exposure and pathogenic agents of exposures, shaping both nutrient and chemical fate and toxicity.^3^ Advancements in molecular and multi-omic tools have enabled exposure signatures to be linked to biological pathways, phenotypes, and disease risk.^4^ Epidemiology provides the population-level framework necessary to translate these molecular insights into evidence relevant for public health, while integrative exposure science connects individual-level measurements to environmental, spatial, and societal determinants. Figure 1 maps this progression from exposure measurement across scales, through causal inference, to prevention and redesign.

**Figure 1:**
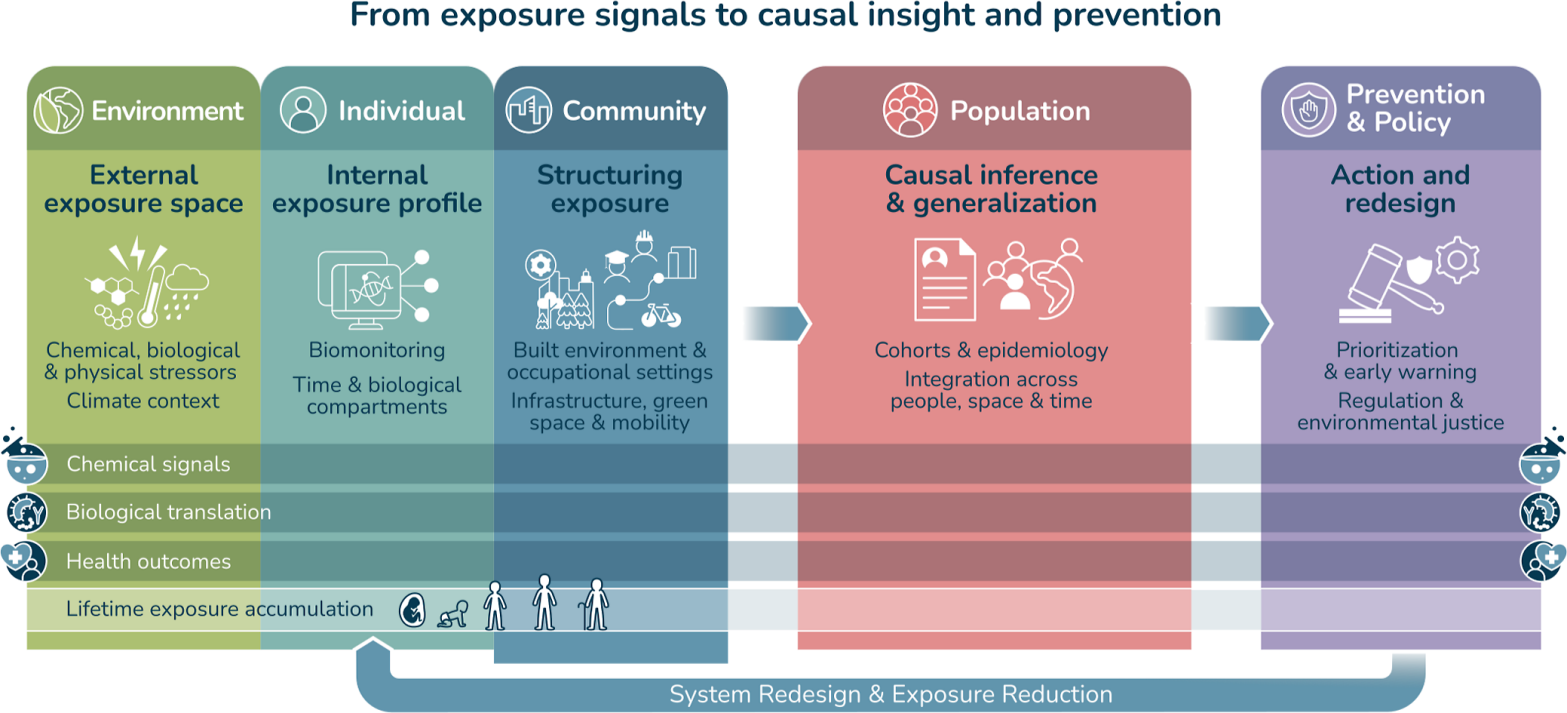
Conceptual framework of the human exposome integrating exposure measurement, causal inference, and prevention across environmental, individual, and population scales.

This *Perspective* posits that the future of environmental health science lies in embracing this continuum, from the environment to human health, rather than treating exposure, biology, and population context as separate domains. By drawing together key advances and emerging frontiers across these interconnected areas, we aim to outline how exposome science can move beyond describing environmental harm toward predicting risk and enabling prevention. In doing so, we highlight the role of *ES&T* not only as a chronicler of environmental problems but also as a platform for building the scientific foundations needed to protect human health over the next 60 years.

### Mapping the Human Chemical Exposome

The advent of high-resolution mass spectrometry (HRMS) has transformed our capacity to interrogate the chemical landscape of human biospecimens. Modern HRMS platforms, particularly those coupling liquid or gas chromatography with time-of-flight or Orbitrap analyzers, routinely detect tens of thousands of molecular features in a single blood or urine sample,^5^ revealing a chemical space of unprecedented breadth and complexity. This technological evolution has catalyzed a paradigm shift from hypothesis-driven quantification of predetermined pollutant panels toward comprehensive, data-driven *chemical cartography* of the human exposome. Rather than focusing on a few hundred priority chemicals, as in traditional biomonitoring programs, exposomics now seeks to systematically map the vast, dynamic molecular terrain shaped by both external exposures and endogenous metabolism. The transition from targeted to nontargeted analysis represents more than an expansion of analytical scope; it fundamentally redefines what we consider measurable exposure. HRMS-based exposomics captures not only parent compounds from environmental, dietary, and occupational sources but also their biotransformation products, endogenous metabolites responding to chemical stress, and previously unrecognized contaminants that evade conventional surveillance. Surveys employing LC-HRMS and GC-HRMS have demonstrated the capacity to simultaneously profile lipophilic persistent pollutants, polar transformation products, and metabolic biomarkers across diverse biological matrices, from serum and urine to breast milk and tissue.^6^ This comprehensive chemical profiling reveals that the internal exposome encompasses a dynamic reservoir far more complex than external exposure inventories would suggest.

Yet the promise of comprehensive chemical measurement confronts a formidable challenge: detection vastly outpaces interpretation. While HRMS platforms can capture millions of spectral features, the majority remain structurally unannotated, representing a chemical “dark matter” whose biological significance remains obscure. Converting these molecular signals into actionable exposure information requires robust strategies for compound identification, source attribution, and functional characterization. Here, advances in computational annotation have proven to be essential. Machine learning algorithms trained on experimental retention time libraries, collision cross-section (CCS) databases derived from ion mobility spectrometry, and in silico fragmentation prediction tools are progressively improving molecular annotation confidence.^7,8^ The integration of CCS values as an orthogonal descriptor, in particular, has enhanced isomer resolution and reduced false-positive identifications when coupled with accurate mass and tandem mass spectrometry data.^9^

Yet a single measurement in a single matrix provides only a fragmentary view of the exposome. Longitudinal biomonitoring reveals significant temporal variability in circulating chemicals, with many exogenous compounds exhibiting limited intraclass correlation coefficients over time,^10^ underscoring the need for repeated measurements to capture chronic exposure patterns. Beyond temporal dynamics, multi-matrix approaches reveal how exposures distribute and transform across biological compartments. Matrix-resolved profiling demonstrates compartment-specific retention, selective transfer across biological barriers, and tissue-specific biotransformation that reshape internal exposure profiles.^11^ For instance, maternal–fetal studies show selective placental retention of certain chemical classes alongside the efficient transplacental transfer of others, illustrating how biological barriers actively filter the fetal exposome during critical developmental windows. Understanding these spatiotemporal distributions is essential for reconstructing exposure histories, identifying windows of vulnerability, and linking external exposures to internal doses at biologically relevant sites.

An important next Frontier in chemical exposomics is the transition toward *functional exposomics*, bridging molecular fingerprints with biological pathways, phenotypes, and disease risk.^12^ This requires moving beyond chemical annotation to establish biologically plausible links between exposure signatures and health outcomes. Integrating untargeted chemical profiling with multi-omics platforms (e.g., transcriptomics, proteomics, metabolomics) enables system-level mapping of how chemical exposures perturb endogenous biochemistry, activate stress response pathways, and modify disease susceptibility.^4^ Such integrated analyses are beginning to reveal exposure-specific molecular phenotypes, offering the potential to develop a molecular epidemiology of exposure in which chemical signatures serve as both biomarkers and mechanistic probes. Multi-omic approaches also reveal interactions between dietary and environmental exposures, as nutrients modulate xenobiotic metabolism pathways.

While HRMS-based biomonitoring enables personalized assessment of individual exposures across time and matrices, many environmental drivers operate at the regional and community scales. Air pollution, water contamination, and socio-economic determinants are inherently spatial and collective phenomena that shape individual exposomes but cannot be fully captured through personal biomonitoring alone. Reconciling molecular precision with a broader environmental context requires integrating personal measurements with environmental surveillance, geospatial modeling, and social determinants of health, mirroring the broader challenge in precision medicine of leveraging individual-level data for both personalized intervention and population-level public health action.

The path forward demands continued innovation across the analytical pipeline: expanding chemical space coverage through orthogonal separation techniques, harmonizing workflows for longitudinal and multi-cohort comparability, and developing open-access reference libraries that democratize structural annotation. But fundamentally, mapping the human chemical and biological exposome-including microbial pathogens, their metabolites, and host–microbiome interactions^13^-is not an end in itself; it is the foundation for a new molecular epidemiology capable of linking environmental chemistry and microbiology to human biology, and ultimately, to prevention.

### Biological Exposures and the Microbial Interface

The exposome encompasses chemical, physical, and biological exposures, with microbial agents and microbial products representing major and integral components of human exposure. Humans can be exposed to microorganisms, pathogens, allergens, nucleic acids, and microbial metabolites, which directly influence health, immunity, metabolism, development, and disease susceptibility. Exposure to microorganisms may result in an altered microbiome,^14^ pathogens cause a broad range of morbidities and mortalities, allergens cause an inflammatory response,^15^ nucleic acids can be incorporated into commensal bacterial genomes,^16^ and microbial metabolites can promote and harm health.^17^ At the same time, exposure to chemical contaminants can alter the human response to microbial agents through immunotoxic effects.^18^

Metrology of biological agents in the environment is in its infancy. Standard measurement methods including quantitative polymerase chain reaction, plaque assays, microscopy-based methods, and immuno-based assays have low accuracy, precision, and repeatability and are time-consuming even when carried out by the most skilled technicians.^19^ These issues can plague efforts to understand factors controlling their distribution, fate, and transport in the environment, challenging a clear understanding of the microbiological exposome. Similarly, an understanding of full suite of microbial chemical metabolites and their potential for exposures and health effects is only recently becoming possible with nontargeted mass spectrometry techniques.

Humans and biological systems, in general, are not passive recipients of various chemical and biological exposures. Host physiologies, including their microbiomes and microbial communities, actively transform, amplify, or buffer chemical and biological contaminants, reshaping resultant exposure profiles. Chemical and biological contaminants can disrupt or transform the host–microbiome system, causing antibiotic resistance, dysbiosis, and metabolic disruption. Host–microbiome–environment interactions form a dynamic biochemical interface where xenobiotics are metabolized, and biological responses are modulated. Recent work demonstrates that microbial communities biotransform environmental pollutants including PFAS,^20^ and climate-driven shifts in microbial ecology are expected to further reshape these exposure dynamics.

Integration of traditional approaches (e.g., epidemiology, risk assessment, animal models) with emerging tools (e.g., metabolomics, proteomics, epigenomics, metagenomics, and transcriptomics) could provide needed insights into better understanding the complex interactions and processes that give rise to the chemical and microbiological exposome and its health effects. Recent advanced experimental tools (organoids, organ-on-chip, exposome-on-chip) may allow for more rapid insights to be made that do not require human subjects or the use of animals.

### Scaling the Exposome: From Personalized Medicine to Community Health

The exposome operates across multiple interconnected scales, from molecular processes within individual cells to regional patterns of environmental contamination and social determinants of health. This multiscale reality creates a productive tension: precision medicine seeks to tailor interventions to individual exposure profiles and genetic susceptibilities, while public health aims to identify collective exposure burdens and implement population-level interventions. Reconciling these perspectives is essential for translating exposome science into both personalized risk assessment and equitable health protection. At the individual level, the human exposome is frequently viewed within the context of precision medicine in which the focus is the individual and with system boundaries that are defined by the human body.^21^ In this view, exposures occur through a variety of pathways (e.g., inhalation, ingestion, and dermal absorption) and at levels that depend on the individual’s home, work/school, or public environment (e.g., indoor air, and drinking water), their diet, and other individual behaviors. Such biological and chemical agents interact with organ systems. As such, the analysis of the human exposome on this personalized scale focuses on the health impacts triggered by environmental exposures and the mechanisms that contribute to disease burden or health outcomes.

As a complement to the individual level, the environmental exposome can also be viewed through a perspective encompassing larger scales, i.e., groups of people (e.g., communities) or geographic regions.^22^ At this population scale, many environmental drivers of exposure—such as urban air pollution, municipal water quality, and neighborhood built environment characteristics—are inherently collective and shape individual exposure profiles through shared infrastructure and regional conditions. This perspective yields different types of information, such as distributions of chemical exposure levels expressed as averages and percentiles. Evaluations of exposures across distances that encompass groups or communities would need to account for regional-scale environmental metrics such as climate, water, and air quality data. Examples of such data are mass fluxes (e.g., air to surface deposition) and solute concentrations averaged over disparate time resolutions. Assessments of exposures across large scales include the analysis of exposure across multiple groups, such as neighborhoods within a large metropolitan region or multiple homes that vary in the quality of ventilation, water, and other components of the built system. Critically, exposure burdens are systematically stratified across communities with marginalized populations disproportionately facing elevated exposures to environmental hazards. Large scale assessments are essential for identifying vulnerable subpopulations and rooting out inequalities.

Bridging individual-level molecular precision with population-scale spatial and social contexts poses significant analytical and infrastructural challenges. Integrating high-resolution biomonitoring data with GIS-based exposure models, environmental surveillance, and sociodemographic information requires harmonized data standards, spatiotemporal modeling frameworks, and rigorous privacy protections. Yet this integration is precisely what enables exposome science to move beyond describing individual exposures toward understanding the structural determinants that stratify exposure burdens across populations and, ultimately, how they can be addressed through policy and intervention.

Linkage of routine environmental and public health surveillance data sets across spatial and temporal scales can reveal population-level health impacts of variation in the human exposome. In the public health field, this approach is often referred to as *environmental public health tracking*.^23^ For example, researchers have linked individual-level blood lead surveillance data for a large cohort of young children with information on household tap water sources and subsequent behavioral outcomes in adolescence, yielding new insight into the long-term risks of lead exposure from drinking water.^24,25^ That work demonstrated that early life exposure to lead in drinking water was associated with increased risk of juvenile delinquency many years later.^25^ In another example, investigators linked routine emergency department intake data with community-scale measures of drinking water microbiological quality, providing evidence of associations between exposure to microbial contaminants and acute gastrointestinal illness (AGI).^26^ That study estimated that 7.3% of emergency department visits for AGI could be attributed to microbiological contamination of drinking water. Beyond water quality, spatial analysis integrating air quality monitoring with health surveillance has revealed how neighborhood-level exposures, including traffic density, proximity to industrial facilities, and lack of green space, are associated with disparities in childhood asthma, cardiovascular events, and birth outcomes, thereby identifying communities where environmental interventions would yield the greatest health benefits.^27^ Collectively, these examples illustrate how the linkage of exposome-relevant data to public health surveillance systems can illuminate the combinations of exposures for which large-scale interventions may yield the greatest population-level health benefits. Ultimately, individual-level precision and population-level public health are not competing paradigms but complementary approaches, each essential for protecting human health in the exposome era. Realizing this potential will require GIS-linked exposome profiles for large populations—developed with rigorous protections against the disclosure of personal health information—and sustained collaboration with public health agencies that manage surveillance data systems.

### From Data to Causality

The exposome era is defined not only by data abundance but also by profound structural complexity: millions of correlated exposure features, heterogeneous biological readouts, and sparse sampling across critical life stages. Such complexity exceeds the capacity of conventional regression-based epidemiology and requires analytical paradigms that explicitly integrate imbalance, sparsity, and prior biological knowledge. Exposome-scale inference must move beyond “big data” toward effective data, leveraging small-sample learning, transfer learning, and structured representations to extract causal signals from fragmented evidence.^28^ At the same time, purely data-driven exposomics risks remaining descriptive unless constrained by hypothesis-driven designs, critical exposure windows, and mechanistic validation.^29^

A central obstacle to causal inference is that low-level, chronic, and mixed exposures remain weakly observed and poorly validated. A potential approach to address this knowledge gap is a cohort-to-molecule strategy in which epidemiologically identified chemical mixtures are reconstructed and tested across human-relevant dose ranges in organoids and animal models, anchoring subtle exposures to specific molecular pathways and benchmark doses.^30^ Similarly, real-world extracts from human blood have been used to identify additive neurotoxic effects at concentrations far below individual chemical thresholds.^31^ This finding challenges single-chemical causality assumptions and illustrates why reliance on high-exposure evidence systematically underestimates risk from typical exposures.^32^ Causality thus emerges from the strategic integration of epidemiology, experimental biology, and model-enabled inference that rebalances evidence across the full exposure continuum. Linking exposome profiles with public health surveillance can further strengthen hypothesis generation and precision public health.^33^

Exposome-wide association studies (ExWAS) demonstrate that aggregate poly-exposomic models explain phenotypic variation comparable to polygenic scores, while individual exposures account for <1% of variance,^34,35^ an empirical finding that motivates systematic discovery at scale. Recent advances in various statistical learning models (e.g., AI and Bayesian inference) can enable integrative frameworks that synthesize observational, experimental, and mechanistic evidence. Bayesian hierarchical designs allow uncertainty-aware integration of heterogeneous evidence, while causal discovery algorithms constrain learning through directed acyclic structures informed by biology. Coupled with transfer learning and foundation models, these approaches can propagate information from data-rich domains to sparse, disease-specific settings, thereby improving causal robustness.^36,37^ Multilayer knowledge network approaches further strengthen causal inference by embedding chemicals, molecular targets, pathways, and health outcomes within unified graphs.^36^ Multilayer knowledge networks integrate chemicals, targets, pathways, and health outcomes into unified graph structures, enabling the identification of plausible mechanisms and potential intervention nodes.^38^ Graph-based models and explainable AI methods can trace multi-hop paths from exposure to outcome, transforming predictions into testable mechanistic hypotheses.

Looking forward, digital twins and dynamic modeling frameworks represent a promising, though still evolving, paradigm for personalized exposure and risk prediction.^39^ In the exposome context, digital twins can be conceptualized as individualized, data-driven representations that integrate time-resolved external exposures, internal biomonitoring, and physiological or molecular states to predict individualized trajectories of risk across the life course. A foundation model demonstrates that self-supervised training models can predict long-term metabolic and cardiovascular risk beyond conventional biomarkers.^40^ This highlights how AI foundation models transform continuous, high-dimensional data into transferable representations for an individual health risk assessment. To ensure policy relevance, AI-enabled exposome modeling must adhere to FAIR principles of Findable, Accessible, Interoperable, and Reusable. Fragmented validation chains and inconsistent metadata undermine regulatory trust; standardized ontologies, persistent identifiers, and machine-readable metadata are essential for interoperability and independent evaluation. AI systems intended for health risk prioritization must move beyond black-box prediction, but should be structured inference tools embedded within causal, mechanistic, and data-aware frameworks.^41^

Finally, causal inference and decision support depend on bridging molecular-scale evidence with population-scale exposure patterns and, when possible, public health surveillance data. Integrating individual-level molecular data with regional exposure models, such as GIS-based pollution mapping, remote sensing, and emission inventories,^42,43^ can enable “source-target” relationships to be contextualized within realistic spatial and socioeconomic settings. HRMS, integrated with AI, enables quantitative source fingerprinting by resolving both target and suspect chemicals within complex mixtures.^44,45^ Beyond source allocation, we can consider that HRMS can further expand exposome-scale chemical coverage, linking structured exposure signatures to biological pathways and strengthening causal inference through mechanism-informed, mixture-aware frameworks. These data can be further linked to public health surveillance data to gain further insights. Such cross-scale integration is essential for translating causal understanding into actionable, geographically targeted public health and environmental policies.

### Translating Knowledge into Prevention and Policy

The exposome framework represents more than an analytical paradigm; it is a foundation for transforming how environmental health science informs regulation, prevention, and intervention. As exposome science matures from mapping diverse exposures and biological responses toward causal inference and predictive modeling, it offers an opportunity to shift environmental health policy from reactive risk management to proactive prevention strategies. This translation is particularly urgent in the context of global chemical production, pathogen emergence, and environmental degradation, which collectively continue to outpace the regulatory capacity.

Traditional regulatory frameworks operate through compound-by-compound risk assessment, evaluating individual chemicals in isolation despite evidence that real-world exposures involve complex mixtures, biological agents, and multi-stressor environments operating across biological, spatial, and social scales.^46^ Integrating multi-omics data into regulatory science enables hazard identification and risk prioritization based on biological signatures rather than solely on external concentration thresholds.^47^ Effect-based early warning systems that combine high-throughput bioassays with nontargeted chemical and biological profiling can identify emerging contaminants, novel antimicrobial resistance genes, and changing mixture toxicity before widespread harm occurs,^48^ providing regulators with the lead time needed for preventive action.

Beyond surveillance, the exposome reframes chemistry itself as a discipline of prevention. Green chemistry embodies this shift from mitigation to proactive design.^49^ Rather than treating toxicity as an unavoidable consequence of chemical innovation, green chemistry principles prioritize intrinsic safety: designing molecules that are benign by design, incorporating degradability, and minimizing bioaccumulation. Exposome data directly inform this innovation by identifying chemical classes and structural features associated with adverse biological signatures, thereby guiding the development of safer alternatives with reduced bioactivity and environmental persistence.

The built environment represents a critical intervention point where exposome science translates to population-level health benefits. Across urban, suburban, and rural settings, built environments concentrate diverse environmental stressors (air pollution, noise, heat islands, chemical mixtures, and microbial exposures) alongside social determinants that modulate vulnerability.^50^ Exposome-informed preventive design and planning integrate health considerations into transportation systems, green space allocation, water infrastructure, and building standards, recognizing that the built environment fundamentally shapes exposure profiles.^51^ Translating exposome knowledge into prevention also demands the address of systemic inequities. Social, economic, and environmental conditions systematically stratify exposure burdens to both chemical and biological agents, creating disparities that standard risk assessments often fail to capture.^52^ Environmental justice requires not only reducing aggregate exposures but also dismantling the structural processes that concentrate hazards in marginalized communities. Ultimately, the utility of exposome science for policy depends on transparent, accessible, and FAIR data systems that enable evidence synthesis across stakeholders.

### Future Vision: From Describing Damage to Designing Health

The next six decades will witness environmental health science transforming from a discipline that documents harm to one that predicts risk and intervenes before exposure-related disease manifests. The critical bottleneck will shift from measurement capacity to interpretation and decision-making. As articulated in recent international exposome initiatives,^53–55^ the convergence of comprehensive exposure assessment, systems biology, artificial intelligence, and precision medicine and public health creates unprecedented opportunities to reimagine population health. Integrating exposomics with genomics will enable precision health strategies, where individual susceptibilities, exposure histories, and biological responses inform personalized intervention and population-scale interventions. But precision without interpretation becomes an end in itself. Wearable sensors, satellite-based environmental monitoring, and digital health records now enable continuous, dynamic exposome tracking.^56^ These technologies become transformative only when embedded in transparent causal frameworks that clarify when evidence warrants action. “Exposome intelligence” must prioritize decision relevance over statistical novelty, guiding intervention rather than endlessly refining exposure metrics. Technological sophistication alone will not suffice. The defining challenge is determining which signals justify action under uncertainty. The exposome’s promise lies equally in exposing the structural, social, and environmental determinants that stratify disease risk across populations. Achieving this requires political will to dismantle systemic processes that concentrate hazards in marginalized communities, not just better measurement tools.^57^ Equity is not ancillary but central: exposome science must inform prevention, not merely document disparity.

The maturation of exposome science will redefine environmental health research: from risk assessment to proactive design and from pollution monitoring to health-centered design of environments, products, and cities. This demands valuing the clarity of inference and responsible action over the completeness of measurement. Journals like *ES&T* must not only inform advances but also set standards for interpretability, causal reasoning, and policy relevance. The defining question for the next 60 years is not how much we can measure but how wisely we act on what we already know.
